# Hepatoprotective effects of berberine on carbon tetrachloride-induced acute hepatotoxicity in rats

**DOI:** 10.1186/1749-8546-5-33

**Published:** 2010-09-18

**Authors:** Yibin Feng, Ka-Yu Siu, Xingshen Ye, Ning Wang, Man-Fung Yuen, Chung-Hang Leung, Yao Tong, Seiichi Kobayashi

**Affiliations:** 1 School of Chinese Medicine, The University of Hong Kong, 10 Sassoon Road, Pokfulam, Hong Kong SAR, China; 2 Department of Medicine, The University of Hong Kong, Queen Mary Hospital, Pokfulam Road, Hong Kong SAR, China; 3 Department of Chemistry and Open Laboratory of Chemical Biology of the Institute of Molecular Technology for Drug Discovery and Synthesis, Faculty of Science, The University of Hong Kong, Pokfulam Road, Hong Kong SAR, China; 4 Department of Medical Laboratory Science, Faculty of Health Sciences, Hokkaido University, Kita-12, Nishi-5, Kita-ku, Sapporo, Japan

## Abstract

**Background:**

Berberine is an active compound in *Coptidis Rhizoma *(*Huanglian*) with multiple pharmacological activities including antimicrobial, antiviral, anti-inflammatory, cholesterol-lowering and anticancer effects. The present study aims to determine the hepatoprotective effects of berberine on serum and tissue superoxide dismutase (SOD) levels, the histology in tetrachloride (CCl_4_)-induced liver injury.

**Methods:**

Sprague-Dawley rats aged seven weeks were injected intraperitoneally with 50% CCl_4 _in olive oil. Berberine was orally administered before or after CCl_4 _treatment in various groups. Twenty-four hours after CCl_4 _injection, serum alanine aminotransferase (ALT) and aspartate aminotransferase (AST) activities, serum and liver superoxide dismutase (SOD) activities were measured. Histological changes of liver were examined with microscopy.

**Results:**

Serum ALT and AST activities significantly decreased in a dose-dependent manner in both pre-treatment and post-treatment groups with berberine. Berberine increased the SOD activity in liver. Histological examination showed lowered liver damage in berberine-treated groups.

**Conclusion:**

The present study demonstrates that berberine possesses hepatoprotective effects against CCl_4_-induced hepatotoxicity and that the effects are both preventive and curative. Berberine should have potential for developing a new drug to treat liver toxicity.

## Background

Liver damage induced by carbon tetrachloride (CCl_4_) involves biotransformation of free radical derivatives, increased lipid peroxidation and excessive cell death in liver tissue [1,2]. This model of CCl_4_-induced liver injury has been widely used in new drug development for liver diseases.

Berberine is a plant alkaloid present in many medicinal herbs, such as *Hydrastis canadensis*, *Coptidis Rhizoma*, *Berberis aquifolium*, *Berberis aristata *and *Berberis vulgaris *[3]. *Coptidis Rhizoma *(*Huanglian*), which is rich in berberine, exhibited hepatoprotective effects on CCl_4_-induced liver injury via scavenging the peroxidative products [4]. Antioxidative effects of *Coptidis Rhizoma *and its major active ingredient berberine against peroxynitrite-induced kidney damage were demonstrated *in vitro *and *in vivo *[5]. Previous studies reported that berberine inhibited inflammation [6] and low-density lipoprotein (LDL) oxidation [7]. Other studies found that berberine was a candidate drug for Alzheimer's disease [8] and cancer [9]. Berberine exhibited no curative action on CCl_4_-induced liver injury whereas serum alanine aminotransferase (ALT) and aspartate aminotransferase (AST) levels were ameliorated after berberine treatment [10]. It is interesting that we showed in our previously study *Coptidis Rhizoma *exhibits curative effect of CCl_4_-induced liver injury in rats, which is discrepant to the reference reports since berberine is considered as the major active compound in *Coptidis Rhizoma *[4]. To clarify the gap and discrepancy among the above reports, it is necessary to do a more systematic and comprehensive study on hepatoprotective effects of bererbine in CCl4-induced acute liver toxicity.

The present study aims to examine the preventive and curative effects of berberine on liver injury and serum, tissue superoxide dismutase (SOD) levels and the tissue histology.

## Methods

### Drugs and chemical reagents

Berberine, CCl_4 _Heparin, Phenobarbital and olive oil were obtained from Sigma (USA). ALT and AST test kits were purchased from Stanbio (USA). SOD assay kit was obtained from Dojindo Laboratories (Japan).

### Animals

Male Sprague-Dawley rats aged 7 weeks weighing 230-270 g were obtained from the Laboratory Animal Centre of the University of Hong Kong. Animals were allowed to acclimate for two days; they were fed with standard pellet diet and water *ad libitum *at 20-25°C under a 12 hour light/dark cycle. Food was withdrawn one day before the experiment but water continued to be provided.

All animal handlings and experiment protocols complied with the guidelines of the Laboratory Animal Centre of the University of Hong Kong. Animals were processed (including drug treatment and sacrifice) in accordance with the international guidelines for laboratory animals.

### CCl_4_-induced acute liver damage model

48 animals were divided into six groups, namely Group 1: control group, Group 2: CCl_4 _control group, Group 3: low dose treatment group (post-treated with berberine, 80 mg/kg), Group 4: medium dose treatment group (post-treated with berberine, 120 mg/kg), Group 5: high dose treatment group (post-treated with berberine, 160 mg/kg) and Group 6: preventive dose treatment group (pre-treated with berberine, 120 mg/kg). Each group contained eight animals. Rats from Groups 2 to 6 were intraperitoneally (ip) injected with CCl_4 _at a dose of 1.0 ml/kg as a 50% olive oil solution while Group 1 received 1.0 ml/kg of olive oil. Berberine was suspended in distilled water at concentrations of 80, 120 and 160 mg/kg which were orally administered through a stomach tube to rats in Groups 3 to 5 respectively after six hours of CCl_4 _treatment. Rats in Group 6 were orally administered with berberine (120 mg/kg) twice daily for two days before CCl_4 _treatment. The CCl_4 _control group (Group 2) was orally administered with distilled water of the equivalent volume.

Twenty-four (24) hours after CCl_4 _administration, the animals were anesthetized with ketamine/xylazine mixture (ketamine 67 mg/kg, xylazine 6 mg/kg, ip). Blood samples were collected by cardiac puncture, placed in heparinized tubes and centrifuged at 3000 × g (Eppendorf, Germany) for 10 minutes to obtain sera which were used to determine SOD and to test ALT and AST activities.

Immediately after blood collection, the animals were sacrificed by an overdose of pentobarbitone (Phenobarbital 200 mg/kg, ip). The liver of each rat was promptly removed and used to determine the tissue level SOD and for further histopathological study.

### Serum ALT and AST analyses

ALT and AST activities in serum samples were measured with Stanbio kits and a UV-rate auto-analyzer (Hitachi 736-60, Japan).

Values of the serum ALT and AST activities were derived according to the 'absorptivity micromolar extinction coefficient' of NADH at 340 nm and were expressed in terms of unit per liter (U/L). One unit per liter was defined as the amount of enzyme required to oxidize one μmol/L of NADH per minute.

### Measurement of serum SOD

Serum SOD was determined according to the technical manual of the SOD assay kit-WST (Dojindo Laboratories, Japan).

Briefly, the assay kit utilized the mitochondrial activity producing a water-soluble formazan dye upon reduction with the superoxide anion. The rate of the reduction with a superoxide anion was linearly related to the xanthine oxidase (XO) activity and was inhibited by SOD. Thus, the inhibition rate of XO activity determined by a colorimetric method was used to reflect the serum SOD levels in this study.

### Histopathological analysis

Liver samples were immediately collected and fixed in 10% buffered formaldehyde solution for a period of at least 24 hours before histopathological study. Samples were then embedded in paraffin wax with Automatic Tissue Processor (Lipshaw, USA) and five-micron sections were prepared with a Leica RM 2016 rotary microtome (Leica Instruments, China). These thin sections were stained with hematoxylin and eosin (H&E) and mounted on glass slides with Canada balsam (Sigma, USA). Degrees of liver damage were estimated as described before[4] under a light microscope (Leica Microsystems Digital Imaging, Germany) and images were captured with a Leica DFC 280 CCD camera (Leica, Germany) at original magnification of 10 × 10. The grades of liver damage in different groups were assigned in numerical scores (scale from 0 to 6).

### Statistical analysis

Data were presented as mean and standard deviation (SD). When one-way ANOVA showed significant differences among groups, Tukey's *post hoc *test was used to determine the specific pairs of groups that were statistically different. A level of *P *< 0.05 was considered statistically significant. Analysis was performed with the software SPSS version 16.0 (SPSS Inc, USA).

## Results

### Effects of berberine post-treatment on serum ALT and AST activities

**Figure 1:**
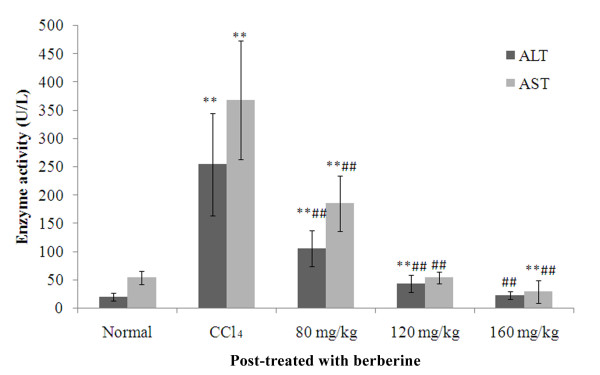
**Effects of berberine post-treatment on serum ALT and AST activities in rats with CCl_4_-induced acute liver damage**. **P <0.001 against normal control; ^##^*P <*0.001 against CCl_4 _control. ALT: 80 mg/kg vs 120 mg/kg, F = 3.1, *P *= 0.004; 120 mg/kg vs 160 mg/kg, F = 51.0, *P *= 0.144; 80 mg/kg vs 160 mg/kg, F = 4.1, *P *< 0.001; AST: 80 mg/kg vs 120 mg/kg, F = 5.3, *P *< 0.001; 120 mg/kg vs 160 mg/kg F = 1.0, *P *= 0.315; 80 mg/kg vs 160 mg/kg, F = 6.3, *P *< 0.001; mean (SD), *n *= 8.

Effects of berberine on serum ALT and AST activities in rats from various treatment groups are shown in Figure 1. After 24 hours of CCl_4 _treatment, the serum ALT and AST activities increased significantly (ALT: F = 11.5, P < 0.001; AST: F = 12.8,*P *< 0.001). Serum ALT and AST activities in berberine co-treatment groups of 'Low dose', 'Medium dose' and 'High dose' decreased significantly in a dose-dependent manner (ALT: Low: F = 7.3, *P *< 0.001; Medium: F = 10.3, *P *< 0.001;High: F = 11.3, *P *< 0.001; AST: Low: F = 7.4, *P *< 0.001; Medium: F = 12.8, *P *< 0.001; High: F = 13.8, *P *< 0.001 when compare when CCl_4 _group). Both medium and high doses of berberine suppressed the ALT and AST activities up to or lower than the level in normal rats (ALT: Medium: F = 1.2; *P *= 0.254; High: F = 0.1, P = 0.906; AST: Medium: F = 0.0, *P *= 0.999; High: F = 1.0, *P *= 0.316 when compared with normal group)

### Effects of berberine post-treatment on serum SOD activity

**Figure 2:**
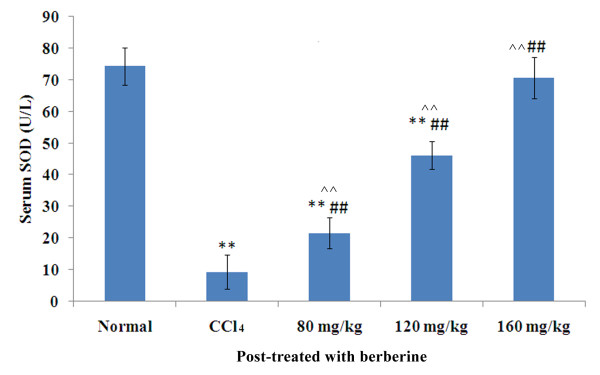
**Effects of berberine post-treatment on serum SOD activity in rats with CCl_4_-induced acute liver damage**. **P <0.001 against normal control; ^##^*P <*0.001 against CCl_4 _control and ^^*P *< 0.001 among three different dosages; mean (SD), *n *= 8. SOD: 80 mg/kg vs 120 mg/kg, F = 9.0, *P *< 0.001; 120 mg/kg vs 160 mg/kg, F = 8.9, *P *< 0.001; 80 mg/kg vs 160 mg/kg, F = 18.0, *P *< 0.001; mean (SD), *n *= 8.

Effects of berberine on serum SOD activity in various treatment groups are shown in Figure 2. After 24 hours of CCl_4 _treatment, serum SOD activity decreased significantly (F = 23.8, *P *< 0.001) and the serum SOD level in berberine co-treatment groups of 'Low' and 'Medium' and 'High' increased significantly in a dose-dependent manner (Low: F = 4.5, *P *< 0.001; Medium: F = 13.5, P < 0.001; High: F = 22.5, *P *< 0.001 when compared with CCl_4 _group). The high dose group (160 mg/kg berberine) showed normal SOD level (F = 1.4, *P *= 0.173 when compared with normal group) which was the best among the three berberine treatment groups.

### Effects of berberine pre-treatment on serum ALT and AST activities

**Figure 3:**
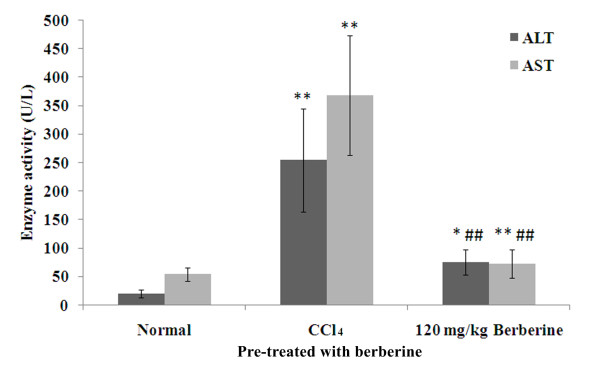
**Effects of berberine pre-treatment on serum ALT and AST activities in rats with CCl_4_-induced acute liver damage**. *P < 0.01 vs normal control; **P <0.001 vs normal control; ^##^*P <*0.001 vs CCl_4 _control; mean (SD), *n *= 8.

Effects of berberine pre-treatment on serum ALT and AST activities in rats treated with CCl_4 _at a dose of 1.0 ml/kg are shown in Figure 3. Serum ALT and AST activities in rats pre-treated with berberine were significantly lower than those in rats treated with CCl_4 _(ALT: F = 8.8, *P *< 0.001; AST: F = 12.0, *P *< 0.001).

### Effects of berberine pre-treatment on serum SOD activity

**Figure 4:**
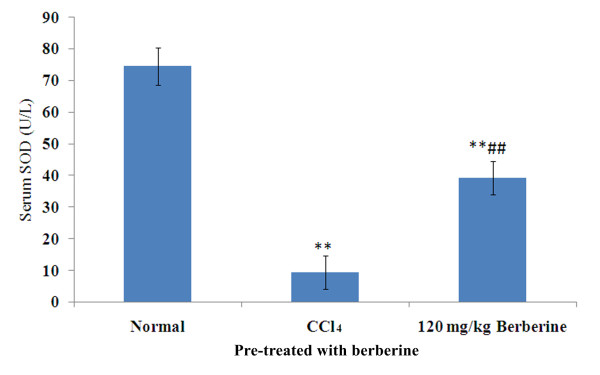
**Effects of berberine pre-treatment on serum SOD activity in rats with CCl_4 _-induced acute liver damage**. **P < 0.001 vs normal control; ^##^*P <*0.001 vs CCl_4 _control; mean (SD), *n *= 8.

Effects of berberine pre-treatment on serum SOD activity of rats are shown in Figure 4. While the serum SOD activity in rats from berberine pre-treatment was significantly lower than that in normal rats (F = 12.9, *P *< 0.001), it was much higher than that in rats treated with CCl_4 _(F = 10.9, *P *< 0.001).

### Histology

**Figure 5:**
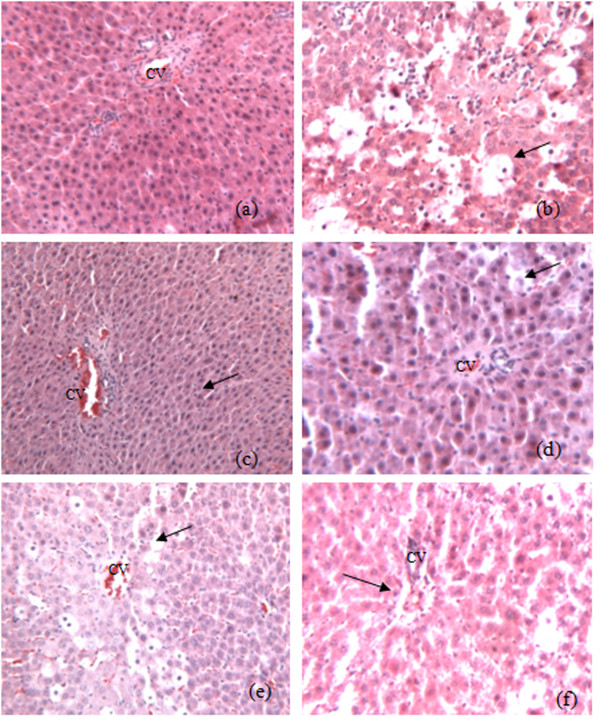
**Photomicrography of liver sections of rats**. **a**. liver sections of normal rats treated with olive oil vehicle only; **b**. liver section of the control rat treated with CCl_4 _only; **c**. liver section of the CCl_4_-treated rat post-treated by berberine at 160 mg/kg; **d**. liver section of the CCl_4_-treated rat post-treated by berberine at 120 mg/kg; **e**. liver section of the CCl_4_-treated rat post-treated by berberine at 80 mg/kg; **f**. liver section of the CCl_4_-treated rat pre-treated by berberine at 120 mg/kg twice daily for two days (H&E stain, original magnification ×100).

Results from the histological studies were in agreement with the measured activities of serum enzymes. There were no abnormalities or histological changes in the livers of normal rats (Figure 5a). Severe hepatocyte necrosis, inflammatory cells infiltration, fatty degeneration, hemorrhage and hydropic degeneration were found in rats 24 hours after CCl_4 _treatment (Figure 5b). Vacuole generation and microvascular steatosis were also observed. Post-treatment of berberine at 160, 120 and 80 mg/kg reduced the severity of CCl_4_-induced liver intoxication (Figures 5c, d and 5e). Fatty change, necrosis and lymphocyte infiltration were improved in the histological sections of berberine post-treated rats. Pre-treatment of berberine before CCl_4 _intoxication also attenuated the hepatic damage induced by CCl_4 _(Figure 5f). These results indicated the effects of berberine against CCl_4_-induced acute liver damage in a dose-dependent manner (Table 1).

**Table 1:** Microscopic observation on protective and preventive effects of berberine against CCl_4_-induced acute liver damage in rats (*n *= 8).

Group	Fatty degeneration Mean (SD)	Vacoulisation Mean (SD)	Nuclei Mean (SD)	Hepatocyte necrosis Mean (SD)	Inflammatory cells infiltration Mean (SD)	Central vein and portal triad Mean (SD)	Combined score Mean (SD)
Normal	0.6 (0.3)	0.3 (0.2)	1.3 (0.3)	0.5 (0.1)	0.7 (0.3)	2.2 (0.6)	1.2 (0.3)
CCl_4_	5.5 (1.2)**	4.8 (0.4) **	0.3 (0.2) **	5.7 (1.9) **	5.5 (1.5) **	0.4 (0.2) **	4.7 (0.9)^##^
Post-treated with berberine							
80 mg/kg	3.2 (1.6) ^##^	2.5 (0.7) ^##^	1.7 (0.3) ^##^	2.2 (0.4) ^##^	2.7 (1.1) ^##^	1.2 (0.5) ^##^	3.0 (1.3) ^## ^
120 mg/kg	1.7 (1.3)^##^	1.8 (0.2) ^##^	1.7 (0.5) ^##^	1.6 (0.8) ^##^	1.8 (0.2) ^##^	2.1 (0.6) ^##^	1.8 (0.9) ^##^
160 mg/kg	1.4 (0.9) ^##^	1.2 (0.4) ^##^	1.4 (0.4) ^##^	1.2 (0.5) ^##^	1.1 (0.4) ^##^	1.5 (0.8) ^##^	1.1 (0.8) ^##^
Pre-treated with berberine							
120 mg/kg	2.1 (1.3) ^##^	2.3 (1.6) ^##^	1.0 (0.7) ^## ^	2.0 (1.4) ^##^	1.5 (0.6) ^##^	2.5 (0.4) ^##^	2.8 (1.4) ^## ^

**P < 0.001 when compared with normal group; ^##^*P *< 0.001 when compared with CCl_4 _group. The *P *values higher than 0.001 were denoted after the means.

## Discussion

In the present study the CCl_4 _treatment alone and post-treatment after 24 hours caused severe acute liver damage in rats, as evidenced by increased serum ALT and AST activities and a decreased serum SOD level (Figures 1 and 2). This phenomenon was confirmed by histological changes (Figures 5a and 5b). Different from previous report (which showed that berberine has no curative effect on acute liver damage) [10], results from this study suggest that post-treatment with berberine may protect liver function. In addition, the histological sections of rat livers post-treated with berberine in Figure 5c-e showed reduced incidence of liver lesions, hepatocyte swelling, leukocyte infiltrations and necrosis induced by CCl_4 _(Figures 5a and 5b). Histological evidence from this study supports the effectiveness of berberine to treat liver damage caused by CCl_4_.

Hwang *et al. *reported that berberine exhibited antioxidant property by its ability to quench free radicals of 1,1-diphenyl-1-picrylhydrazyl, decrease the leakage of lactate dehydrogenase and ALT and prevent the formation of malondialdehyde induced by t-BHP [11]. Janbaz and Gilani reported that post-treatment with berberine (4 mg/kg) after CCl_4_-induced hepatotoxicity exhibited no effect in reducing hepatic damage [10]. Sun *et al*., however, reported that berberine protected liver injury evidenced by decreased ALT and AST activities and that berberine's action was focused on liver fibrosis in CCl_4_-induced rats [12]. The apparent discrepancy between the two studies may be due to the dosages, animal species and animal models used. The present study found that berberine had both preventive and curative effects on CCl_4_-induced liver damage. Moreover, our findings suggest that dosages may be an important factor for curative effects of berberine. The dosage (4 mg/kg) used by Janbazour *et al. *was far below the effective dosage (80-160 mg/kg) reported in this study, which was determined according to our clinical experience [13] and was similar to the dosage reported by Sun *et al. *[12].

Pre-treatment of berberine significantly decreased both serum ALT and AST activities elevated by CCl_4_-induced hepatoxicity while serum SOD level significantly decreased (Figures 3 and 4). These results demonstrate the preventive hepatoprotective effects of berberine against liver damage induced by CCl_4_, further supported by the histological changes (Figure 5f).

## Conclusion

The present study finds that berberine possesses hepatoprotective activities against CCl_4_-induced hepatotoxicity in a dose-dependent manner. The heptoprotective activities are both preventive and curative. These findings were further supported by the histological changes in the liver. Berberine should be a lead for developing new drugs to treat drug/chemical-induced liver toxicity.

## Abbreviations

ALT: alanine aminotransferase; AST: aspartate aminotransferase; CCl_4_: Carbon tetrachloride; CRAE: *Coptidis Rhizoma *aqueous extract; H&E: hematoxylin and eosin; ROS: reactive oxygen species; SOD: superoxide dismutase; XO: xanthine oxidase; CCD: Charge-coupled device

## Competing interests

The authors declare that they have no competing interests.

## Authors' contributions

YF designed the study, conducted the experiments, analyzed the data and drafted the manuscript. KYS, XY and NW conducted the experiments, collected the data and helped draft the manuscript. MFY, CHL, YT and SK interpreted the data and revised the manuscript. All authors read and approved the final version of the manuscript.
